# Editorial: Adaptation of traditional crop cultivars to climate change in terms of nutritional aspects

**DOI:** 10.3389/fnut.2024.1427068

**Published:** 2024-05-23

**Authors:** Márta Ladányi, Anna Divéky-Ertsey, László Csambalik

**Affiliations:** ^1^Department of Applied Statistics, Institute of Mathematics and Basic Science, Hungarian University of Agriculture and Life Sciences, Budapest, Hungary; ^2^Department of Agroecology and Organic Farming, Institute of Rural Development and Sustainable Production, Hungarian University of Agriculture and Life Sciences, Budapest, Hungary

**Keywords:** wild crop relatives, biodiversity, sustainability, plant population heterogeneity, traditional crop varieties, nutritional quality

A global decrease of crop plant nutritional values is experienced worldwide, partly due to the breeding preferences of the last decades, which focused mainly on yields and tolerance to plant pests and diseases. These parameters are crucial when food security is concerned, however, tasteless raw produces can raise consumer ignorance and the feedback has an impact on agricultural production systems as well. In comparison with old traditional cultivars and their wild relatives, new modern plant varieties perform worse in terms of organoleptic features, especially of volatile components content. Both open-field and protected plant cultivation are influenced by the recent phenomena of climate change; adaptability is the key for future crop production. This can be achieved by technological developments and by preparing the plant materials to harsh environmental conditions through breeding efforts.

Heterogeneity is of substantial importance when food security is concerned. The decreasing number of cultivated crop species and varieties means higher exposure to the threats of changing environmental conditions. Diverse plant populations have higher resilience and adapting ability to unfavorable biotic and abiotic conditions. Traditional plant cultivars are valuable genetic sources in terms of higher nutritional properties as well as of yield security due to their high adaptability to regional conditions. The utilization of these cultivars as breeding materials could enhance both poor nutritional properties and adaptability of commercial varieties. Efforts are required when traditional cultivars are to be integrated into modern agricultural systems due to their limitations in terms of yield quantity and of storage life. However, their added value is complex, consisting of traditional aspects, regional culinary importance, environmental adaptability, and nutritional richness, therefore both *in-situ* conservation and *ex-situ* utilization should be considered.

Diversification is the key component of sustainable agriculture, which can be interpreted in multiple levels from the land to the fork. Measures to maximize diversity affects the whole food system. The inclusion of crop wild relatives and underutilized species or genotypes into cultivation can be an effective way to increase both the diversity of agricultural lands and of the human diet as well, directly impacting human wellbeing and health. Utilization of agricultural diversity is the common point of the works of Fukalova et al., Elouadi et al., Udhaya Nandhini et al., and Stoleru et al., who published their results within the frames of this Research Topic in Frontiers in Nutrition.

Crop species level diversification is the first step for sustainable plant production, which has direct and indirect advantages on the environment. In several countries, wild edible plants are the part of the regional agricultural heritage and the living elements of the local cuisine. Abundantly growing low-demanding wild plants are often considered as weeds, without any knowledge about their nutritional or organoleptic properties. In the Mediterranean region, plants, such as pigweed, chickweed, sowthistle, and wall rocket are popular food sources and condiments, due to their rich aroma provided by their high volatile constituent content. The work of Fukalova et al. sheds the light to these plants by analyzing the volatile profile of several wild edible plants, identifying 37 different compounds, mainly monoterpenoids and benzoids. The abundance of volatiles in the investigated plants highlights their potential to be utilized for culinary use and for enriching the species diversity of monotonous agricultural lands.

With the re-introduction of traditional crop varieties, the variety level diversification of sustainable agriculture can be achieved. These long-forgotten varieties are often excluded from agricultural practice, regardless to their possibly valuable traits encoded in their genetic pool. There is a worldwide growing interest in traditional rice varieties experienced, which is triggered by their possibly high nutritional and pharmaceutical value. The work of Udhaya Nandhini et al. therefore focused on five traditional rice varieties and analyzed their metabolic compositions, identifying over 149 metabolites grouped into 34 chemical classes. The investigated five rice varieties were grouped into two main clusters, highlighting the high biochemical complexity of traditional rice metabolome, and supporting future exploitation of genetic materials in nutritional targeted breeding.

Diversification in human diet requires the thorough knowledge of food materials. Biofortification, the targeted breeding process with the aim to produce nutrient-rich varieties, can directly serve the enrichment of human dietary requirements. The study of Elouadi et al. is a good example of utilizing wild crop relatives and genetic materials in breeding for the development of nutritionally rich new varieties. They targeted those hull-less genotypes with high β-glucan content and investigated its extent of synthesis in the function of different environments using a genotype plus genotype × environment (GGE) model. Their work identified the environmental influence of the investigated genetic materials, outlining the best genotype—environment combination within the range of the experiment.

The increasing focus on the importance of diverse living rhizosphere is experienced due to the worldwide degradation of soils. Intensive, chemical input-based agricultural management strategies, including chemical fertilizer and pesticide use, all contribute to the microbial depopulation of soils. However, soil microbial activity is the key for the proper nutrient supply of plants, which can be supported by a technological shift in nutrient management. In their study, Stoleru et al. investigated the impact of different nutrient supply regimes on the yield, mineral and phytochemical properties of sweet pepper. They concluded, that the use of organic and biological fertilizers has a positive effect on most traits studied, highlighting the potential underlying in the diversification of soil microbiome.

## Author contributions

ML: Writing – review & editing, Supervision, Conceptualization. AD-E: Writing – review & editing, Supervision, Conceptualization. LCs: Writing – review & editing, Writing – original draft, Conceptualization.

